# Immunologically Inert Nanostructures as Selective Therapeutic Tools in Inflammatory Diseases

**DOI:** 10.3390/cells10030707

**Published:** 2021-03-23

**Authors:** Laura Talamini, Eiji Matsuura, Luisa De Cola, Sylviane Muller

**Affiliations:** 1 CNRS-University of Strasbourg, Biotechnology and Cell Signaling, Illkirch, France/Strasbourg Drug Discovery and Development Institute (IMS), Institut de Science et D’Ingénierie Supramoléculaire, 67000 Strasbourg, France; talamini@unistra.fr; 2 Neutron Therapy Research Center, Collaborative Research Center, Department of Cell Chemistry, Okayama University, Okayama 700-8558, Japan; eijimatu@md.okayama-u.ac.jp; 3 Department of Molecular Biochemistry and Pharmacology, Istituto di Ricerche Farmacologiche Mario Negri IRCCS, 20156 Milan, Italy; luisa.decola@marionegri.it; 4 Department of Pharmaceutical Sciences (DISFARM), University of Milano, 20122 Milan, Italy; 5 Fédération Hospitalo-Universitaire OMICARE, Fédération de Médecine Translationnelle de Strasbourg, Strasbourg University, 67000 Strasbourg, France; 6 University of Strasbourg Institute for Advanced Study (USIAS), 67000 Strasbourg, France

**Keywords:** therapeutic carriers, nanoparticles, drug targeting, immunomodulation, neutrophils, NETosis

## Abstract

The current therapies based on immunosuppressant or new biologic drugs often show some limitations in term of efficacy and applicability, mainly because of their inadequate targeting and of unwanted adverse reactions they generate. To overcome these inherent problems, in the last decades, innovative nanocarriers have been developed to encapsulate active molecules and offer novel promising strategies to efficiently modulate the immune system. This review provides an overview of how it is possible, exploiting the favorable features of nanocarriers, especially with regard to their immunogenicity, to improve the bioavailability of novel drugs that selectively target immune cells in the context of autoimmune disorders and inflammatory diseases. A focus is made on nanoparticles that selectively target neutrophils in inflammatory pathologies.

## 1. Introduction

The immune system is made of a complex panel of proteins, cells, organs and effectors that continuously protect our organism from foreign infectious agents (viruses, bacteria, parasites) and aggressive agents (such as toxins). In fact, its main function is to permanently differentiate our own constituents, which by definition are massively “visible”, from external possibly invading elements. It maintains cell homeostasis and prevents the accumulation of possibly deleterious dead cells that can be toxic. It is responsible for the so called “immune surveillance” that favors the ignorance of self (tolerance) and fights the non-self [1]. The immune system consists of two general lines of defense that generate innate and adaptive immune response. Innate response is rapid and can be triggered without selective events that underlie adaptive immunity. It is induced by phagocyte cells, inflammation serum proteins and cell receptors that signal and release chemokines and cytokines to clean off pathogens from the organism, for example. When the innate immune system is overwhelmed, a second line of defense is activated, the adaptive immunity, which leads to the activation and recruitment of immune cells (B and T lymphocytes) that mediate specific humoral and cellular response, respectively [2]. Therefore, the immune system represents an essential checkpoint for the host survival by generating various “tools” to get rid of pathogenic invaders and also to induce immunologic memory and tolerance to self-antigens. Despite the fact that this system is tightly regulated, aberrant activation of its components can occur. This excessive response can lead to a worsening of inflammation and possible autoimmune reactions, which can evolve towards acute or chronic pathologies, such as sepsis, asthma, obesity and type 2 diabetes (T2D), cardiovascular diseases (e.g., atherosclerosis, cardiac ischemia/reperfusion), metabolic diseases, cancer, autoimmune diseases [e.g., intestinal bowel diseases (IBD), multiple sclerosis (MS), rheumatoid arthritis (RA), systemic lupus erythematosus (SLE)] and neurodegenerative diseases [3]. In this context, a scheduled therapeutic intervention is necessary to restore the homeostasis and the normal function of the immune system by its activation or suppression (immunotherapy). For example, immunostimulatory therapies could be used for the treatment of cancer and infectious diseases, in which the activation of immune response could detect and eliminate non-self-antigens and establish memory effects. Conversely, in diseases like atherosclerosis, MS, RA, SLE, and diabetes, in which there is an over-activation of immune responses, the treatment should downregulate or immunomodulate (redirect) the immune reactions and raise immune tolerance. 

In the last years, despite the ever-increasing development of new therapeutics, a significant fraction of these has encountered several obstacles once administered to patients. This is the case of current medications such as some immunosuppressant and anti-inflammatory drugs (dexamethasone, methotrexate, rapamycin) [4,5], and more recently therapeutic antibodies (e.g., anti-cytokines such as anti-TNFα, anti-IL-1, anti-IL-6, or anti-receptor and signaling molecules) [6,7,8], biologics (including DNA and other genetically-engineered proteins) and peptides [9]. Although these molecules and biopharmaceuticals show efficacy and alleviate devastating symptoms, their long-term interest is often mitigated by inappropriate intrinsic properties. The main drawbacks are their low specificity toward the desired target, and as consequence, their spread throughout the body and accumulation in off-target organs. Further, their low selectivity imposes the use of large amounts of such active substances that adds a limiting parameter due to their toxicity at high dose. In addition, long-term therapy protocols, usually required for improving their efficacy, can lead to a severe systemic toxicity or immunodeficiency in case of accumulation into the body. Finally, some of these active molecules also show poor solubility in water, rapid metabolism and strong susceptibility to endogenous enzymes that overall also hamper their bioactivity [10]. 

Based on these premises, nanotechnologies and in particular the exploitation of nanoparticles (NPs) carrying active molecules, represents a useful strategy to improve the therapeutic effect of current therapies and overcome many obstacles due to the treatment method. The ultimate goal is to design NPs that are safe, immunologically inert (i.e., furtive) and versatile in terms of active drug release at the scheduled time and the intended site of action and, of course, are completely eliminated by the body.

## 2. On the Importance of Developing Immunologically Furtive Nanostructures

An important feature of a therapeutic that can alter its performance in terms of pharmacokinetics and efficacy is its own potential immunogenicity. State-of-the-art therapies of inflammatory arthropathies, for example, rely on the use of biological agents (e.g., therapeutic monoclonal antibodies to TNF-α or IL-6). These drugs are very effective in some patients, even when treated over the long term, whereas they exhibit no effect on others especially because of the production of anti-drug antibodies (ADAs). ADA titers are sometimes very high and negatively impact target engagement of the soluble drug in patients. These ADAs can thus block or neutralize the drug. Worse yet, treated patients can even develop clinically significant anaphylactic infusion-related reactions or hypersensitivity reactions. Recent studies show a correlation between the ADAs plasma levels, the drug dose and the efficacy of treatment. Using nanostructures to encapsulate the drug can efficiently solve this problem. However, this protective system has also to be immunologically inert, otherwise known as “furtive”. Any NP must remain undetectable by the innate immune system and just acts as a genius carrier that targets the drug it protects to the adequate organ or tissue. This is of particular importance for repeat dosing of therapeutic drugs, where immune system memory may limit effectiveness of the drug product. Developing NPs of furtive antigenicity is thus decisive for protecting patients during the time of their treatment and potentially also for new cycles of infusion carried out several years later, and to limit the loss of drug efficacy. To this regard, attention should be paid to the surface functionalization of the NPs [11], and any substance that could be adsorbed at the NP surface when injected in tissues (skin, muscle) or via the intravenous way. In particular the molecules absorbed on the NP surface can either act directly as immunomodulators or, due to the repetitive structural pattern, induce unexpected recognition and response, as in the case of polyethylene glycol (PEG)ylated NPs that could induce an anti-PEG immune response and immunological memory, which did not occur in the absence of PEG [12]. The formation of protein corona [13,14] should also be controlled since the presence of specific protein adsorbed on the particle surfaces can induce or inhibit immune response [15,16].

## 3. Nanotechnology and Immunotherapy

Nanotechnology has many definitions, but in general, it is defined as “the intentional design, characterization and production of materials, structures, devices, and systems by controlling their size and shape in the nanoscale range (1 to 100 nm)” [17]. The application of nanomaterials for medical purposes is commonly known as nanomedicine. In the last decades, scientists have been able to synthesize NPs with different sizes [18] and shapes [19,20], porous [21], solid [22] or capsules [23] and possessing different chemical compositions and degrees of degradation. The tiny size of NPs allows them to be “small” enough to penetrate inside cells and subcellular bodies, and “big” enough to load and transport therapeutic molecules. It, therefore, comes as no surprise that in the nanomedicine context, NPs represent a potent source of interest as immune tools. Additionally, the presence of functional groups on NP surface allows the binding with suitable moieties for ad hoc target [24]. In this scenario, the advancement of nanomedicine may greatly help the pharmacokinetics and the target of many active molecules, thus potentially increasing their therapeutic profile. Compared to traditional or biologic drugs administrated under a free form, several studies have pinpointed the advantageous properties of NPs. These features include: (i) improved solubility of hydrophobic molecules; (ii) prolonged half-life in the bloodstream avoiding enzymatic degradation; (iii) increased passage across biological barriers thought size-, shape- and charge- depended manner; (iv) focused target toward tissues or organs due to functional decorations on NP shell avoiding the spread of cargo in off-target compartments; (v) controlled cargo release at a precise dosage by stimuli responsive NPs; (vi) co-delivery of synergistically active molecules to achieve superior therapeutic efficacy adjusting the drug dosage, and (vii) co-loading of drug with diagnostic agents [25]. 

**Figure 1:**
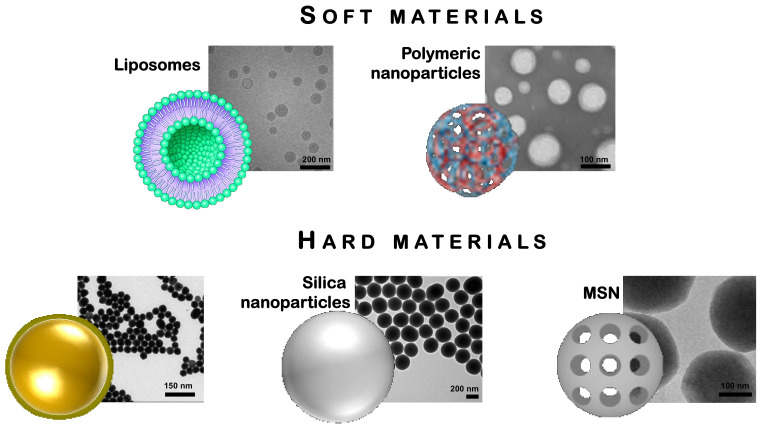
Liposomes, polymeric nanoparticles (NPs), gold NPs, silica NPs and mesoporous silica NPs (MSNs) shown as examples of nanoparticles used for therapeutic purposes [26,27,28,29].

For the treatment of immune disorders, NPs could be used as immunomodulators and more precisely, they could be exploited with a double role by activating or suppressing the immune system. Below, we present an overview of the different types of NPs designed to promote immune tolerance against chronic inflammation and autoimmune diseases (Figure 1). We insist on the fact that the particle shape can play a crucial role in the targeting and recruitment of specific cells, which in turn influences the uptake of the drug the particle brings.

**Figure 2:**
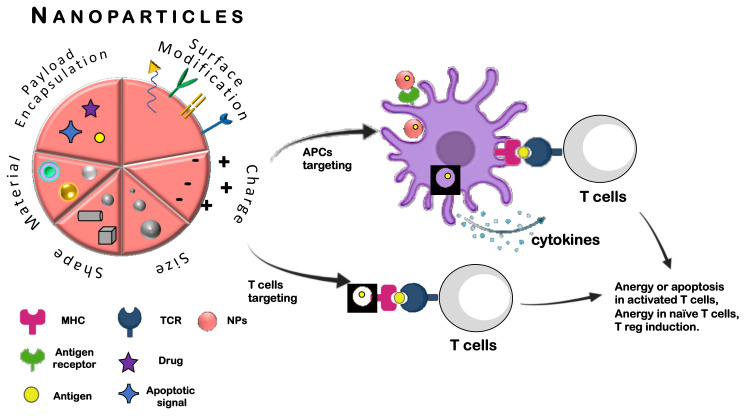
Schematic representation of nanoparticle-based strategies for antigen delivery and tolerance induction by targeting antigen presenting cells (APCs) and by directly targeting T cells.

In the literature, two major approaches based on the application of NPs have been described, namely (i) by targeting antigen-presenting cells (APCs) [30], and (ii) by directly targeting the autoreactive lymphocytes [31] as depicted in Figure 2. The first way of intervention involves APCs such as dendritic cells (DCs), macrophages and B cells that mediate the cellular immune response by processing and presenting antigens via major histocompatibility complex (MHC) molecules to the receptor of T cells (TCR). According to the type of signal that stimulates APCs, different ways of antigen presentation occur via MHC I or MHC II molecules that present epitopes to CD8^+^ T and CD4^+^ T cells, respectively [32]. In the absence of stimulatory molecules, APCs can present the antigen and drive to the inhibition of T cells promoting a status of anergy and/or tolerance [33].

Several reports have pointed out the positive results for tolerance generation mediated by APC for the treatment of MS, autoimmune encephalomyelitis and type-1 diabetes (T1D). One strategy is to employ NPs that mimic apoptotic bodies, thus activating the pathway of elimination and generating tolerance. Some examples are the incorporation of phosphatidylserine (PS) [34], scavenger receptor as macrophage receptor with collagenous structure (MARCO) [35] or antigenic peptides [36] in the NP structure in order to favor the uptake by APCs and to allow the tolerogenic antigen presentation. Further studies aiming at evaluating the effect of NP physiochemical features (e.g., size and shape) with the immune cells showed that an immune tolerizing effect can be obtained through the alteration of NP geometry [37]. The insert of apoptotic signal into NPs is not the only example to achieve the tolerogenic state. Another approach is for example to co-encapsulate in the core of NPs immunosuppressive drugs (e.g., rapamycin, dexamethasone, methotrexate, vitamin D), plasmid DNAs (pDNA) encoding for anti-inflammatory cytokines, small interfering RNA (siRNA) or antisense nucleotides, for example, to induce the downregulation of pro-inflammatory molecule expression [38]. 

The second approach of targeting is the use of appropriate functionalized NPs toward autoreactive lymphocytes (e.g., T cells) thus bypassing the activation of APCs [39]. T cells mediate tolerance through activation of mediators that in turn suppress the autoreactivity of T cells themselves. A successful example has been the use of peptide-major histocompatibility (p-MHC) complex to induce tolerance and enhance the therapeutic activity in a broad spectrum of liver autoimmune diseases [40].

## 4. A Focus on Nanoparticles and NETosis

**Figure 3:**
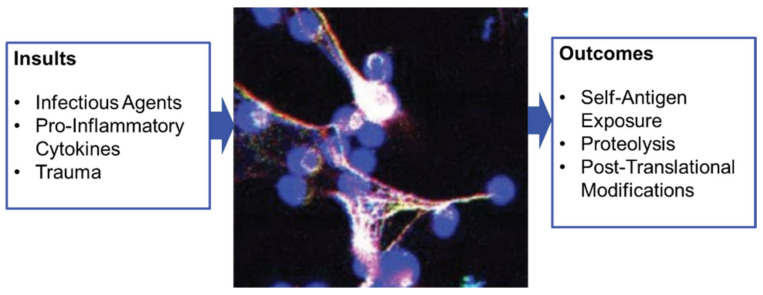
NETosis, a regulated form of neutrophil cell death that contributes to the host defense against pathogens and is also linked to various inflammatory diseases. The cells shown in this figure are primary human neutrophils exposed to A23187 ionophore and stained for DNA (blue) and extracellular histone H1 (yellow).

Recently, an important cell subset of the immune innate system, namely neutrophils, has attracted special attention in the area of therapeutic applications of nanostructures. A few studies have effectively described the use of polymeric particles to modulate the behavior of immune cells, more especially for depleting neutrophils that are recruited in excess in inflamed tissues. While neutrophils are potent defenders of our body that specially act to maintain homeostasis in host tissues, in certain circumstances, and especially under chronic autoinflammatory conditions, they can delay resolution or contribute to pathogenic mechanisms of autoimmune diseases [41,42,43,44]. In autoimmune conditions, neutrophils are chronically active and prone to undergo a peculiar form of cell death, called NETosis, which results in the release of a nuclear chromatin meshwork called neutrophil extracellular trap (NET). NETosis is especially involved in SLE in which it is well-documented that NETs released by activated neutrophils form lattices of decondensed chromatin fibers containing intact DNA filaments, histones and neutrophil enzymes [43,45,46]. In predisposed individuals, these macromolecules can generate pathogenic autoantibodies, especially since their immunogenicity is further enhanced by post-translational modifications, such as deimination of arginine residues, that modify their conformation and their interaction with molecular partners and ligands (Figure 3).

Neutrophils are the most abundant white blood cell in humans and mice. Depleting an abnormally raised production of neutrophils in inflamed tissues is a potent therapeutic strategy for avoiding they bolster hyper immunoreactivity, especially self-reactivity. Downregulating excessive NETosis may also be a valuable therapeutic option in certain acute infections in which it can be deleterious. An example of application of a nanostructure-based strategy in this context has been recently proposed to treat SARS-CoV-2-mediated illness. In this work, the authors used DNAse-I-coated melanin-like nanospheres that mitigated sepsis-associated NETosis [47].

Some studies have reported important differences of cell interaction as a function of nanoparticles size, shape and surface chemistry [48,49,50,51]. With regard to neutrophils, it was reported for example that the presence of PEG chains at the surface of polymeric particle affects their phagocytosis by primary human neutrophils, which is increased, or by macrophages or monocytes, which is decreased [52]. Zhang et al. developed a doxorubicin-conjugated bovine serum albumin nanosystem which selectively targets activated neutrophiles via Fcγ receptors and promotes their apoptosis in order to improve the therapeutic efficacy in inflammatory diseases in which this pathway is altered [53]. Recent ex vivo and in vivo experiments also indicated that independent of the material type, rod-shaped polymeric particles are selectively internalized by neutrophils compared to monocytes [54]. This example especially illustrates the fact that it is possible nowadays to design nanostructures that would be immunologically neutral and able to target neutrophils selectively with drugs. 

It should be kept in mind, however, that nanomaterial exposure can also induce NETosis [55,56]. This can be a pharmacological advantage to enhance NETosis in certain clinical indications (e.g., when NETosis is defective) but can also be at risk in others when the innate system, especially via neutrophils, is overactivated and NETs are massively released. 

## 5. Traditional Nanomaterials for Immunological Applications

Nowadays, researchers have synthesized and characterized NPs combining different materials, structures, and biological functions for the delivery of drugs, proteins, peptides, oligonucleotides in order to modulate the immune system. On the basis of the material stiffness that assemble NPs, below, we successively describe and discuss the respective interest of soft and hard nanomaterials and also summarized in Table 1.

### 5.1. Polymeric NPs

Polymeric NPs are one of the softest materials used for immune modulation. They are solid and semisolid colloidal particles with a size ranging from 10 to 1000 nm. They can be designed to be biocompatible, biodegradable and safe. The most used include natural polymers such as chitosan, alginate, gelatin and albumin or synthetic materials such as poly (lactide) (PLA), poly (lactide-co-glycol) copolymer (PLGA), poly (ε-caprolactone) (PCL) [57,58]. Polymeric NPs can be used as a drug carrier where the cargo can be absorbed, dissolved, or entrapped into the matrix by polymer dispersion or polymerization. Overall, the term “polymer NPs” encompasses a subcategory of NPs including polymer-drug conjugated, protein-polymer conjugated, polymeric NPs, dendrimers and others [59]. Polymeric carriers show many advantages, such as: (i) a good degree of biodegradability and biocompatibility by design, (ii) well-described method of synthesis adapted to various types of drugs (e.g., hydrophilic or hydrophobic molecules), (iii) selective targeting, (iv) controlled release of the cargo, (v) some protection of the cargo from degradation, (vi) high life-time in the bloodstream, (vii) possibility to modify surface properties to provide “stealthiness” and/or better interactions with biological matrices, and (viii) excellent stability [60]. The main disadvantages are the low degree of loading, the permeability of the polymers, the difficulties in reaching small sizes (below 50 nm) and different morphologies. With some materials, full degradation is also achieved reducing accumulation problems, as for example in the case of NPs made of PLGA polymers, which are completely hydrolyzed into lactic and glycolic acids, which can metabolized via the Krebs cycle into the body and hence, that has received FDA (Food and Drug Administration) and EMA (European Medicines Agency) approval for human use [61].

Significant attempts have been done to target DCs using polymeric NPs. DCs are optimal candidates since they hold a key role in the modulation of immune system and possess high phagocytic capacity. Although it has been demonstrated that the physicochemical features of NPs (e.g., size, surface charge) could enhance the interaction with DCs [62,63], the most effective approach to precisely target DCs would be providing NPs with specific targeting ligand. For example, Cruz et al. built PEGylated PLGA NPs decorated with distinct antibodies against TNF-α family (CD40), integrin (CD11c) and c-type (DEC-205) and loaded with ovalbumin and Toll-like receptor (TLR)-3 and- 7 ligands. Results demonstrated that all targeted NPs induced CD8^+^ T cell responses both in vitro and in vivo [64]. Another strategy to enhance the uptake of polymeric NPs by DCs is the functionalization with mannan (mannose receptor) [65] as carriers to deliver therapeutic oligonucleotides [66]. Mannosylation could be also a target of macrophages. Thus, Deng et al. showed that mannose-modified chitosan NPs loaded with micro (mi)RNA-146b are taken up selectively by intestinal macrophages in the treatment of colitis, resulting in a localized suppression of pro-inflammatory cytokines [67]. Notably, dextran and its derivate act as ligands for macrophages due to the expression of dextran-binding proteins and scavenger receptors on macrophages surface. For this reason, they have received increasing attention in biomedical application as well as they possess remarkable biocompatibility and biodegradability [68]. In a murine model of RA, dextran sulfate NPs showed a higher accumulation in inflamed joints due to an active uptake by macrophages responsible for the inflammation. Moreover, the combination of targeting with the encapsulation of an anti-rheumatic drug significantly improved the therapeutic efficacy compared with free drug [69]. Polymeric NPs with a semi-solid shell can be a reservoir to encapsulate molecules easily degradable if administered alone (e.g., drugs or antigens). Maldonado et al. synthesized PLGA NPs containing a peptide antigen and the immunosuppressive drug, rapamycin. The nanocarrier administration induced the inhibition of T cell activation and generation of T regulatory cells (Treg), inducing the antigen specific tolerance in autoimmune encephalomyelitis (EAE) mouse model [70]. In a model of T1D, a diabetogenic peptide was either entrapped or conjugated to PLGA NPs. In vivo results showed that functionalized NPs were really taken-up by APCs and upregulated the expression of anti-inflammatory cytokines IL-10 and TGF-β. More interestingly, the treatment induced amplification of Treg and restored tolerance in both CD4^+^ and CD8^+^ T cells [71]. In another work, Cappellano et al. reported that polymeric NPs co-loaded with IL-10 and myelin oligodendrocyte glycoprotein (MOG) peptide decreased inflammatory cytokine secretion by T cells in mice with EAE without any cytotoxicity effect [72]. Finally, in our own laboratory, we showed that NPs containing hyaluronic acid (HA) and a 21-mer therapeutic peptide called P140, which is currently evaluated in phase-III clinical trial in lupus patients, were efficient when given via the intra-duodenal route to Murphy Roths large/lymphoproliferative (MRL/lpr) lupus-prone mice [57]. Proteinuria, that appear in these mice with age was delayed and remained less frequent all along the course of the disease. The intra-duodenal administration of HA-P140, a first step for the future design of oral administration of peptide, was also shown to be beneficial on the mortality rate of treated mice. In the control group, the first mice died at 15 weeks of age while in the treated group, the first mouse died at 22 weeks only. At 37 weeks, 100% of non-treated mice and only 50% of mice having received the HA-P140 were dead. These results led us to conclude that intra-duodenal administration of HA-P140 NPs to MRL/lpr lupus mice allows their life expectancy to be lengthened in a significant way.

### 5.2. Liposomes

Liposomes are other carriers largely used in nanomedicine and the first NP-mediated anti-cancer drug approved by FDA [73]. Several studies have demonstrated that liposomes provide a versatile platform for delivery hydrophilic or hydrophobic payloads to immune cells [74,75]. Liposomes are vesicles having one or more concentric lipid bilayers composed of natural or synthetic phospholipids enclosing an aqueous core [76]. Liposomes have been widely used to entrap anti-cancer, anti-inflammatory, anti-fungal, and antibiotic drugs or genes [77] because of their unique properties. They are able to self-assemble in aqueous solution and encapsulate both hydrophilic (core) and hydrophobic (bilayer membrane) therapeutic molecules with high efficacy. The large aqueous core permits to deliver small and macro molecules such as nucleic acids, proteins, imaging agents and at the same time to protect the cargo from undesired degradation, early inactivation or dilution. In addition, liposomes are suitable for ligand conjugation to target specific cells, organ or tissue of interest. Liposomes are biocompatible, biodegradable, and due to their nature, they are considered safe [78]. 

Since it has been largely established that the use of immunosuppressive drug such as corticosteroids, is accompanied by significant side effects, including muscle weakness, osteoporosis, diabetes, and neurodegeneration, the strategy to encapsulate these drugs into a liposome has already become widespread [79]. For example, liposome-based steroidal nano-drug demonstrated its ability to accumulate preferentially in inflamed joints after intravenous administration and thus resulted in a complete remission of inflammatory response in rat arthritis model [80]. A similar formulation proved also positive efficacy in decreasing anti-dsDNA serum antibody levels, proliferation of lymphoid tissue and renal damages, and in prolonging survival in a murine model of lupus [81]. In order to avoid the spread of the cargo around the body, another strategy is to exploit decorated particles to selectively reach the desired site of action. For example, phosphatidylcholine liposomes were explored in antigen-specific therapy for RA by Capini et al. [82]. Phosphatidylcholine liposomes loaded with antigen and lipophilic NF-kB inhibitor suppressed pre-existing immune response in an antigen-specific manner. Such formulation was able to target APCs in situ, suppress the cellular responsiveness to NF-kB and to induce antigen-specific FoxP3^+^ Tregs [82]. Other authors selected ART-1 (CRNADKFPC) peptide decorated liposomes for targeting the immunomodulatory cytokine IL-27 into the joints of arthritic rats. Although IL-27 was already validated as a potent immunomodulatory cytokine, its therapeutic index is compromised by the side effect because of the long-term exposure. Instead, ART-1-IL-27 liposomes showed a higher accumulation into inflamed arthritic joints, and an enhanced therapeutic efficacy reducing any adverse effects of treatment compared with free cytokine [83]. Additionally, it has also been reported that the use of folate as ligand on the surface of liposomes selectively boosts the accumulation in areas of inflammation [84]. New findings have highlighted the targeting of DCs with antigen-containing liposomes as a promising strategy for inducing tolerance in autoimmune diseases. Especially, it was demonstrated that in mice immunized with an antigenic peptide, the administration of liposomes containing both calcitriol and ovalbumin suppressed the expansion of antigen-specific effector T cells and promoted the expansion of antigen-specific regulatory T cells in a mouse model of RA [85]. The same group pointed out that liposomes encapsulating the peptide, BDC2.5mim, and calcitriol induced Tregs expansion in pre-diabetic mice and the liposomal administration at the onset of the disease delayed diabetes progression [86]. Other researchers functionalized phosphatidylserine-liposomes with MOG and they observed that liposomes induced a tolerogenic phenotype in DCs, and arrested autoimmunity, thus decreasing the incidence, delaying the onset and reducing the severity of EAE [87]. In our laboratory, liposomes have also been shown to display favorable properties to deliver viral and peptides of self-antigen [88,89]. For example, by using liposomes associated with a cyclic peptide encompassing residues 139–147 in site A of the hemagglutinin of influenza A virus, we were able to achieve 70% protection of treated mice against an intranasal challenge with the infectious virus [90]. The liposomal preparation was completely synthetic and contained no adjuvant, an advantageous when large-scale programs of vaccination are engaged. 

However, we draw attention to the fact that cross-reacting antibodies can be generated when non-furtive liposomes are used. Such antibodies may possibly give rise to false positive results in diagnostic assays and/or induce side effects in individuals who are prone to develop autoimmunity. They can also generate unwanted effect in case of repetitive administration (vaccination, long-term treatment). Moreover, liposomal structure presents some disadvantages due to their dynamic nature such as the leakage of the loaded molecules, instability, low solubility, fusion of the encapsulated molecules, and phospholipid structure could undergo to oxidation or hydrolysis [76].

### 5.3. Gold NPs

Gold NPs, said AuNPs, are excellent hard nanomaterials, which are exploited in drug delivery, disease diagnosis and therapy [99,100]. AuNPs are easy to synthesize in different shapes and sizes, present a large surface area-to-volume ratio that facilitates their functionalization with several chemical functionalities. The AuNPs are stabilized by a dense shell of thiol and/or amino groups that can also be used, or exchanged, to anchor molecules such as drugs/genes/antibodies (for therapy), radionuclide/imaging probes (for imaging or diagnostics), and biological and chemical sensors (for photodynamic therapy) [93]. AuNPs are considered very stable and immunocompatible [28] even though recently some doubts where raised on their stability in vivo and on the loss of their coating [101]. The use of AuNPs for immunotherapy applications are for example as carrier to target DCs. In an interesting study they have been used in a T1D mouse model to deliver tolerogenic molecule 2-(1’H-indole-3’-carbonyl)-thiazole-4- carboxylic acid methyl ester and proinsulin to induce DCs that promoted Treg differentiation [102]. Important results were also generated in a model of ischemic skeletal muscle injury with AuNPs that were conjugated with IL-4 anti-inflammatory cytokine. The administration of these NPs induced a macrophage repolarization toward a favorable, anti-inflammatory, M2 phenotype and led to muscle improvement and regeneration [103]. A similar approach has been used by coating AuNPs with a hexapeptide (P12) supposed to inhibit TLR signaling in macrophages. P12 underwent lung accumulation and internalization in lung macrophages. It skewed alveolar macrophages toward an M2 profile, reducing thus the infiltration of inflammatory cells and increasing anti-inflammatory cytokine levels in the bronchoalveolar lavage fluid (BALF) and lung tissue in acute lung injury mouse model [104]. 

AuNPs excellently combine optical and thermal properties, which were especially exploited in cancer therapy [105]. The optical properties of AuNPs are based on surface plasmon resonance (SPR). SPR is a process whereby the electrons of gold oscillate coherently with incident light at a specific frequency in response to incoming radiation, causing them to both absorb and scatter light. Following excitation, part of the energy absorbed by AuNPs is emitted in the form of scattered light while the rest of the energy is dissipated as heat [106]. These two properties enable to apply AuNPs for photoimaging and photothermal therapy. The photothermal approach is thus used to obtain localized heating, either to destroy cells or to release drug.

A number of examples of advanced treatments that combine therapeutic compounds and heat for control delivery have been reported in the literature. In the context of RA, for example, Lee et al. coated polymeric methotrexate (MTX)-loaded NPs with a gold half shell and then attached a peptide encompassing the well-known motif RGD—a cell attachment site present in a large number of adhesive extracellular matrix, blood, and cell surface proteins—on gold shell. The authors demonstrated that under generation of heating by NIR (near-infrared) irradiation, the gold shell produced heat that stimulated the release of drug in a higher amount compared to conventional treatment and led to the accumulation of the formulation at the level of inflamed joints [107]. Kang et al. used an alternative approach to remotely control the orientation and the activity of RGD ligand on AuNPs conjugated with a flexible linker to magnetic NPs. Their data demonstrated that upon magnetic field, RGD peptide changed its configuration and elicited a temporal recruitment of macrophages and their M2 polarization [108].

Despite of the advantageous characteristics of AuNPs, this material is not biodegradable and in the last years, therefore, some concerns have questioned about the impact of gold accumulation inside the body and its possible interaction with immune system cells. Further studies are thus needed to better understand the risks and evaluate the harm and benefit of AuNPs-based therapies [94,95].

### 5.4. Silica NPs

Despite the established preclinical potential of NPs as multimodal nanosystems, until now NPs have achieved only moderate translation to the clinical practice. Nowadays, a large number of hard NPs are exploited on trials, in particular for diagnostics purposes. In recent years, silica and organo-silica materials have reach a high degree of sophistication and have gained attention in the field of diagnostics and therapeutic applications [109]. These materials are inert, non-antigenic, and have excellent stability over time. They are easily tunable by size, shape and surface functionalization, and they can be employed as excellent probes following the encapsulation or conjugation with desired molecules [110]. Furthermore, different studies have demonstrated their good biocompatibility and biodegradability. Quite recently, the FDA has recognized amorphous silica as safe [111] and C-dots silica NPs (~7 nm) have been approved for diagnostic application in phase I clinical trial [112]. 

Among the various silica NPs currently investigated, mesoporous silica particles (MSNs) are considered as ideal carrier due to the presence of large and tunable pores into the framework allowing the loading of several molecules as a suitable reservoir for therapeutic or diagnostic purposes [113,114]. Moreover, stimuli-responsive silica particles have been recently prepared to respond and release the cargo upon redox, enzymes, and UV-light [115,116,117]. 

**Figure 4:**
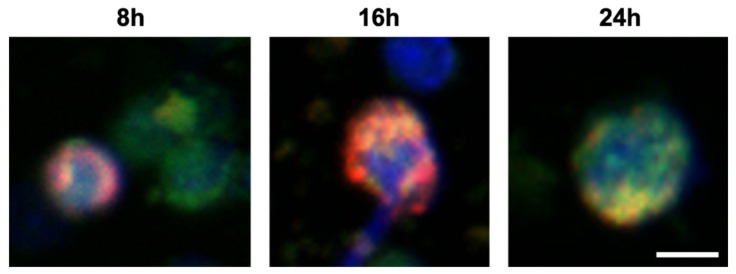
Internalization, ex vivo, of P140-encapsulated silica nanocapsules into murine B cells, as followed by confocal microscopy. Fluorescent images were taken 8 h, 16 h, and 24 h after incubation of P140-NPs with B cells extracted from the liver of Murphy Roths large/lymphoproliferative (MRL/lpr) lupus prone mice. Cells were stained with DAPI (4′,6-diamidino-2-phenylindole) (nuclei, blue), Lysotracker (red) and AF488 P140-nanocapsules (green). Scale bar, 10 μm.

Porous silica NPs were formulated to induce tolerogenic effects by targeting DCs [96]. More especially, this targeting was promoted by the conjugation of NPs with the intracellular adhesion molecule CD209-DC-SIGN and CD11c. Additionally, NPs loaded with rapamycin were shown to increase the number of Tregs in animals [118]. In another formulation, Gan et al. demonstrated that the functionalization with mannose receptor (konjac glucomannan ligand) allowed to target macrophages and induce their polarization to M2 phenotype, promoting the gene expression of arginase 1, MRC and IL-10. In a model of IBD, the administration of mannosylated NPs alleviated the colitis symptoms [119]. Recently, it was reported an interesting example of controlled delivery system based on MSNs to reduce the symptoms of RA. Zhang et al. formulated MSNs functionalized with a cell penetrating peptide and loaded with both miR-30-5p inhibitor or Triptolide, a toxic drug. The innovative way is represented by MSNs stuffed with a light-sensitive material. Upon laser irradiation, the combination therapy with MSNs significantly affected the overactive immune system in a mouse model of RA [120]. MSNs can be combined with other materials to generate a composite system able to respond to external stimuli. For instance, Kim et al. anchored silica NPs with manganese ferrite and ceria to obtain scavenger NPs for reactive oxygen species and to produce O_2_ in the context of RA. Moreover, the loading with MTX showed the development of a system that released the drug in a sustained manner [121]. Finally, MSNs could be inserted in a multimodal system to improve both the selectivity of the target and the release of the payload. For instance, in the context of periodontal diseases in which the site of action is hard to reach, Liu and colleagues used a multifunctional system based on nanofibrous spongy microspheres as scaffold to deliver therapeutic molecules. Inside the scaffold, the authors encapsulated miRNA-based polymeric NPs and MSNs loaded with growth factors (IL-2/TGF-β). In vivo, the release of growth factors promotes the recruitment of T cells, and their transformation toward a Treg phenotype is achieved by the simultaneous delivery of both miR-10s and growth factors [122]. Silica NPs also act an immunomodulator itself on inflamed skin. In a model of allergic contact dermatitis Palmer et al. reported that the acute topical exposure of amorphous SiNPs 20 nm in size decreased the epidermal hyperplasia, inflammatory cytokine release, T cell infiltration and the skin swelling [123]. An example of internalization of the encapsulated therapeutic peptide P140 into lupus B cells is shown in Figure 4. 

## 6. Nanotechnologies as Sensor Devices

As described above, nanoparticle-based therapy offers multiple benefits compared to the conventional treatments. Another further advantage to apply such tiny particles is their application as sensing devices. Shrinking their size, NPs acquire unique properties such as electrochemical or optical features, which are not present in their bulk form, thus offering a significant improvement over conventional diagnostic assays [124]. In numerous therapeutic areas, and especially in the case of cancer but also in the field of autoimmune diseases, the diagnosis relies on the identification of disease-associated hallmarks. In this context, NPs can be advantageously used as biosensors for designing more sensitive, specific and rapid diagnostic tests. Thus, for example, the SSB/La protein, an autoantigen which is associated with several autoimmune diseases (Sjögren’s syndrome, SLE), has been immobilized into a porous silicon matrix and used as such, at very low concentration, for testing patient’s sera on this functionalized surface [125]. Other hard NPs, or quantum dots (QDs), have also been used for the detection or measurement of selected proteins, antibodies or nucleic acids present in various fluids [126,127]. 

**Figure 5:**
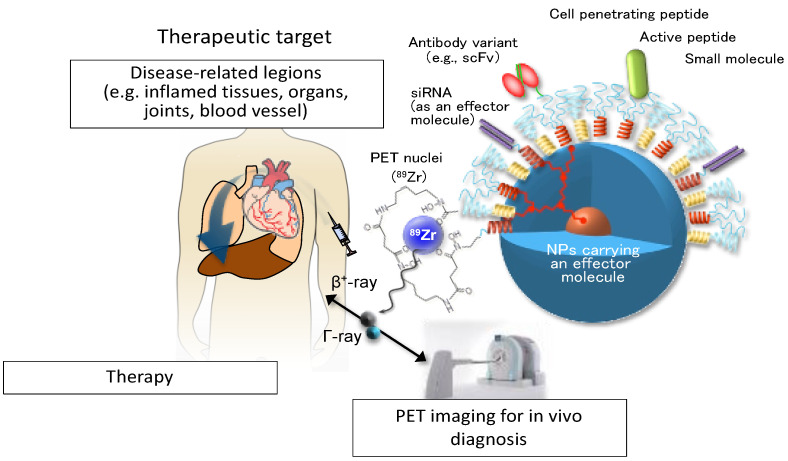
A conceptual representation of an NPs-based theranostic device. NPs carrying an effector molecule such as covalently attached peptide, a small molecule with pharmacological properties, a siRNA presented individually in a single copy or in multiple combinations, can be delivered to selected disease-specific targets. Enhanced or more selective targeting features may be provided using a full antibody or a single-chain variable fragment (scFv) of a specific antibody. Disease condition can be simultaneously monitored by position emission tomography (PET) imaging, for example. For additional details, see [135].

“Smart” nanoparticles have also been engineered to respond to exogenous and endogenous stimuli and acting as sensing devices. The application of stimuli triggers the alternation or the disruption of NPs resulting in active molecules release both on demand and in a targeted manner [128]. Various endogenous stimuli have been exploited from the microenvironment features associated with pathological conditions. They include variations of pH level, redox environments (ROS) or an enhanced concentration of specific enzymes, for example. Exogenous stimuli that have been applied are light, temperature changes, ultrasounds, magnetic and electric field, for example [129]. From several years the most attractive NPs with semiconductor properties were QDs due to their resistance against photobleaching and narrow emission spectra. However, they are quite toxic, limiting their application in vivo. An example of less toxic and promising tool is based on silicon and mesoporous silica NPs in which the external surface can be anchored with diverse imaging agents such as small fluorophores and radionuclides to improve multiple imaging techniques [130,131]. Although these NPs have been widely developed for the detection of tumor mass and treatment of cancer [132,133,134,135], their physical and biological properties could also be extremely valuable in the context of autoimmune diseases, for example to track the localization of damaged tissues in vivo and to monitor the effectiveness of a new therapy (Figure 5).

## 7. Conclusions

Approaches to modulate the innate and adaptive immune system, especially neutrophils reactivity, by NPs have received a lot of attention in the last years not only in the treatment of cancer but also to regulate immune dysfunctions and in particular to generate tolerance in certain diseases in which an aberrant activation of the immune system occurs. The numerous types of NPs represent a versatile toolbox, both as such and as bioinspired carriers of bioactive molecules, thanks to their small size, their shape that can be adapted to the needs and their tunable physicochemical properties. Striking developments are under evaluation based on innovative strategies. This includes, for example, plant extracts in culture to produce innocuous gold NPs, which could be used as an eco-friendly alternative to the chemical methods that produce hazardous by-products [136]. 

This last decade, the application of nanotechnology has achieved important goals in enhancing the efficacy of standard immunosuppressive therapy and improving the selectivity to the target in the treatment of autoimmune and chronic inflammation [137]. The recent findings of Safari et al. [54] highlight the innovative perspectives that are possible henceforth to target neutrophils and NETosis or other cell type, by changing the shape of NPs. This technological breakthrough is central to the development of safer and more efficient therapeutic strategies. In this context, designing immunologically furtive nanostructures is equally decisive. The design of such structures that are not seen by the immune system should also convince the authorities and patients of their inherent harmlessness. Certain nanomaterials are effectively questionable and should be treated with caution in therapeutic options [138]. In the context of diagnostics, the development of antigenically neutral and inert nanostructures is also central to generate automated systems able to analyze thousands of physiological fluids extremely rapidly and in simple conditions. This is crucial in the case of epidemics or environmental catastrophes for instance. Future investigations in these directions will certainly bring tremendous developments.

## Figures and Tables

**Table 1:** Advantages and disadvantages of selected types of nanoparticles.

Type of NPs	Advantages	Disadvantages	Ref
Natural polymer NPs	Biocompatible, degradation products safe and non-toxic, low price	Low water solubility	[91,92]
Co-polymer NPs	Biocompatible, safe and non-toxic, good mechanical properties	Low rate of degradation, poor drug encapsulation	[60,91]
Liposomes	Biocompatible, non-toxic, flexible, improved pharmacokinetics effect of cargo	Low stability, short half-life, leakage and fusion of cargo, oxidation and hydrolysis reactions, may trigger immune reactivity	[76,78]
AuNPs	Easy preparation and functionalization, large surface area, good contrast agent	Low degradability, not biocompatible, poor safe	[28,93,94,95]
Silica NPs	Large surface area, biocompatible and biodegradable, controllable porosity	Low stability, formation of aggregates, hemolysis	[96,97,98]

## References

[1] Parkin J., Cohen B. (2001). An Overview of the Immune System. Lancet.

[2] Chaplin D.D. (2010). Overview of the Immune Response. J. Allergy Clin. Immunol..

[3] Fang R.H., Zhang L. (2016). Nanoparticle-Based Modulation of the Immune System. Annu. Rev. Chem. Biomol. Eng..

[4] Li P., Zheng Y., Chen X. (2017). Drugs for Autoimmune Inflammatory Diseases: From Small Molecule Compounds to Anti-TNF Biologics. Front. Pharmacol..

[5] Tabas I., Glass C.K. (2013). Anti-Inflammatory Therapy in Chronic Disease: Challenges and Opportunities. Science.

[6] Meier F.M.P., Frerix M., Hermann W., Müller-Ladner U. (2013). Current Immunotherapy in Rheumatoid Arthritis. Immunotherapy.

[7] Dinarello C.A. (2019). The IL-1 Family of Cytokines and Receptors in Rheumatic Diseases. Nat. Rev. Rheumatol..

[8] Du F.H., Mills E.A., Mao-Draayer Y. (2017). Next-Generation Anti-CD20 Monoclonal Antibodies in Autoimmune Disease Treatment. Autoimmun. Highlights.

[9] Wilhelm M., Wang F., Schall N., Kleinmann J.-F., Faludi M., Nashi E.P., Sibilia J., Martin T., Schaeffer E., Muller S. (2018). Lupus Regulator Peptide P140 Represses B Cell Differentiation by Reducing HLA Class II Molecule Overexpression. Arthritis Rheumatol..

[10] Schäcke H., Döcke W.D., Asadullah K. (2002). Mechanisms Involved in the Side Effects of Glucocorticoids. Pharmacol. Ther..

[11] Barbero F., Russo L., Vitali M., Piella J., Salvo I., Borrajo M.L., Busquets-Fité M., Grandori R., Bastús N.G., Casals E. (2017). Formation of the Protein Corona: The Interface between Nanoparticles and the Immune System. Semin. Immunol..

[12] Park K. (2010). To PEGylate or Not to PEGylate, That Is Not the Question. J. Control. Release.

[13] Lundqvist M., Stigler J., Elia G., Lynch I., Cedervall T., Dawson K.A. (2008). Nanoparticle Size and Surface Properties Determine the Protein Corona with Possible Implications for Biological Impacts. Proc. Natl. Acad. Sci. USA.

[14] Monopoli M.P., Walczyk D., Campbell A., Elia G., Lynch I., Bombelli F.B., Dawson K.A. (2011). Physical-Chemical Aspects of Protein Corona: Relevance to in Vitro and in Vivo Biological Impacts of Nanoparticles. J. Am. Chem. Soc..

[15] Fadeel B. (2019). Hide and Seek: Nanomaterial Interactions With the Immune System. Front. Immunol..

[16] Cai R., Ren J., Ji Y., Wang Y., Liu Y., Chen Z., Farhadi Sabet Z., Wu X., Lynch I., Chen C. (2020). Corona of Thorns: The Surface Chemistry-Mediated Protein Corona Perturbs the Recognition and Immune Response of Macrophages. ACS Appl. Mater. Interfaces.

[17] Kim B.Y.S., Rutka J.T., Chan W.C.W. (2010). Nanomedicine. N. Engl. J. Med..

[18] Zhu X., Vo C., Taylor M., Smith B.R. (2019). Non-Spherical Micro- and Nanoparticles in Nanomedicine. Mater. Horiz..

[19] Zhao Y., Wang Y., Ran F., Cui Y., Liu C., Zhao Q., Gao Y., Wang D., Wang S. (2017). A Comparison between Sphere and Rod Nanoparticles Regarding Their in Vivo Biological Behavior and Pharmacokinetics. Sci. Rep..

[20] Talamini L., Violatto M.B., Cai Q., Monopoli M.P., Kantner K., Krpetić Ž., Perez-Potti A., Cookman J., Garry D., P Silveira C. (2017). Influence of Size and Shape on the Anatomical Distribution of Endotoxin-Free Gold Nanoparticles. ACS Nano.

[21] Gisbert-Garzarán M., Lozano D., Vallet-Regí M. (2020). Mesoporous Silica Nanoparticles for Targeting Subcellular Organelles. IJMS.

[22] Ma K., Gong Y., Aubert T., Turker M.Z., Kao T., Doerschuk P.C., Wiesner U. (2018). Self-Assembly of Highly Symmetrical, Ultrasmall Inorganic Cages Directed by Surfactant Micelles. Nature.

[23] Udoh C.E., Cabral J.T., Garbin V. (2017). Nanocomposite Capsules with Directional, Pulsed Nanoparticle Release. Sci. Adv..

[24] Blanco E., Shen H., Ferrari M. (2015). Principles of Nanoparticle Design for Overcoming Biological Barriers to Drug Delivery. Nat. Biotechnol..

[25] Feng X., Xu W., Li Z., Song W., Ding J., Chen X. (2019). Immunomodulatory Nanosystems. Adv. Sci. (Weinh).

[26] Cipolla D., Blanchard J., Gonda I. (2016). Development of Liposomal Ciprofloxacin to Treat Lung Infections. Pharmaceutics.

[27] Chiesa E., Pisani S., Colzani B., Dorati R., Conti B., Modena T., Braekmans K., Genta I. (2018). Intra-Articular Formulation of GE11-PLGA Conjugate-Based NPs for Dexamethasone Selective Targeting—In Vitro Evaluation. IJMS.

[28] Swartzwelter B.J., Barbero F., Verde A., Mangini M., Pirozzi M., de Luca A.C., Puntes V.F., Leite L.C.C., Italiani P., Boraschi D. (2020). Gold Nanoparticles Modulate BCG-Induced Innate Immune Memory in Human Monocytes by Shifting the Memory Response towards Tolerance. Cells.

[29] Tarpani L., Morena F., Gambucci M., Zampini G., Massaro G., Argentati C., Emiliani C., Martino S., Latterini L. (2016). The Influence of Modified Silica Nanomaterials on Adult Stem Cell Culture. Nanomaterials.

[30] Cifuentes-Rius A., Desai A., Yuen D., Johnston A.P.R., Voelcker N.H. (2020). Inducing Immune Tolerance with Dendritic Cell-Targeting Nanomedicines. Nat. Nanotechnol..

[31] Prosperi D., Colombo M., Zanoni I., Granucci F. (2017). Drug Nanocarriers to Treat Autoimmunity and Chronic Inflammatory Diseases. Semin. Immunol..

[32] Gaudino S.J., Kumar P. (2019). Cross-Talk Between Antigen Presenting Cells and T Cells Impacts Intestinal Homeostasis, Bacterial Infections, and Tumorigenesis. Front. Immunol..

[33] Kishimoto T.K., Maldonado R.A. (2018). Nanoparticles for the Induction of Antigen-Specific Immunological Tolerance. Front. Immunol..

[34] Pujol-Autonell I., Serracant-Prat A., Cano-Sarabia M., Ampudia R.M., Rodriguez-Fernandez S., Sanchez A., Izquierdo C., Stratmann T., Puig-Domingo M., Maspoch D. (2015). Use of Autoantigen-Loaded Phosphatidylserine-Liposomes to Arrest Autoimmunity in Type 1 Diabetes. PLoS ONE.

[35] Getts D.R., Martin A.J., McCarthy D.P., Terry R.L., Hunter Z.N., Yap W.T., Getts M.T., Pleiss M., Luo X., King N.J.C. (2012). Microparticles Bearing Encephalitogenic Peptides Induce T-Cell Tolerance and Ameliorate Experimental Autoimmune Encephalomyelitis. Nat. Biotechnol..

[36] Lutterotti A., Yousef S., Sputtek A., Stürner K.H., Stellmann J.-P., Breiden P., Reinhardt S., Schulze C., Bester M., Heesen C. (2013). Antigen-Specific Tolerance by Autologous Myelin Peptide-Coupled Cells: A Phase 1 Trial in Multiple Sclerosis. Sci. Transl. Med..

[37] Roberts R.A., Eitas T.K., Byrne J.D., Johnson B.M., Short P.J., McKinnon K.P., Reisdorf S., Luft J.C., DeSimone J.M., Ting J.P. (2015). Towards Programming Immune Tolerance through Geometric Manipulation of Phosphatidylserine. Biomaterials.

[38] Dacoba T.G., Olivera A., Torres D., Crecente-Campo J., Alonso M.J. (2017). Modulating the Immune System through Nanotechnology. Semin. Immunol..

[39] Ben-Akiva E., Est Witte S., Meyer R.A., Rhodes K.R., Green J.J. (2019). Polymeric Micro- and Nanoparticles for Immune Modulation. Biomater. Sci..

[40] Umeshappa C.S., Singha S., Blanco J., Shao K., Nanjundappa R.H., Yamanouchi J., Parés A., Serra P., Yang Y., Santamaria P. (2019). Suppression of a Broad Spectrum of Liver Autoimmune Pathologies by Single Peptide-MHC-Based Nanomedicines. Nature Commun..

[41] Kaplan M.J., Radic M. (2012). Neutrophil Extracellular Traps: Double-Edged Swords of Innate Immunity. J. Immunol.

[42] Jorch S.K., Kubes P. (2017). An Emerging Role for Neutrophil Extracellular Traps in Noninfectious Disease. Nat. Med..

[43] Apel F., Zychlinsky A., Kenny E.F. (2018). The Role of Neutrophil Extracellular Traps in Rheumatic Diseases. Nat. Rev. Rheumatol..

[44] Sollberger G., Tilley D.O., Zychlinsky A. (2018). Neutrophil Extracellular Traps: The Biology of Chromatin Externalization. Dev. Cell.

[45] Muller S., Radic M. (2016). Oxidation and Mitochondrial Origin of NET DNA in the Pathogenesis of Lupus. Nat. Med..

[46] Gestermann N., Di Domizio J., Lande R., Demaria O., Frasca L., Feldmeyer L., Di Lucca J., Gilliet M. (2018). Netting Neutrophils Activate Autoreactive B Cells in Lupus. J. Immunol..

[47] Park H.H., Park W., Lee Y.Y., Kim H., Seo H.S., Choi D.W., Kwon H.-K., Na D.H., Kim T.-H., Choy Y.B. (2020). Bioinspired DNase-I-Coated Melanin-Like Nanospheres for Modulation of Infection-Associated NETosis Dysregulation. Adv. Sci. (Weinh).

[48] Champion J.A., Walker A., Mitragotri S. (2008). Role of Particle Size in Phagocytosis of Polymeric Microspheres. Pharm. Res..

[49] Champion J.A., Mitragotri S. (2009). Shape Induced Inhibition of Phagocytosis of Polymer Particles. Pharm Res..

[50] Hu C.-M.J., Zhang L., Aryal S., Cheung C., Fang R.H., Zhang L. (2011). Erythrocyte Membrane-Camouflaged Polymeric Nanoparticles as a Biomimetic Delivery Platform. Proc. Natl. Acad. Sci. USA.

[51] García I., Sánchez-Iglesias A., Henriksen-Lacey M., Grzelczak M., Penadés S., Liz-Marzán L.M. (2015). Glycans as Biofunctional Ligands for Gold Nanorods: Stability and Targeting in Protein-Rich Media. J. Am. Chem. Soc..

[52] Kelley W.J., Fromen C.A., Lopez-Cazares G., Eniola-Adefeso O. (2018). PEGylation of Model Drug Carriers Enhances Phagocytosis by Primary Human Neutrophils. Acta Biomater..

[53] Zhang C.Y., Dong X., Gao J., Lin W., Liu Z., Wang Z. (2019). Nanoparticle-Induced Neutrophil Apoptosis Increases Survival in Sepsis and Alleviates Neurological Damage in Stroke. Sci. Adv..

[54] Safari H., Kelley W.J., Saito E., Kaczorowski N., Carethers L., Shea L.D., Eniola-Adefeso O. (2020). Neutrophils Preferentially Phagocytose Elongated Particles-An Opportunity for Selective Targeting in Acute Inflammatory Diseases. Sci. Adv..

[55] Muñoz L.E., Bilyy R., Biermann M.H.C., Kienhöfer D., Maueröder C., Hahn J., Brauner J.M., Weidner D., Chen J., Scharin-Mehlmann M. (2016). Nanoparticles Size-Dependently Initiate Self-Limiting NETosis-Driven Inflammation. Proc. Natl. Acad. Sci. USA.

[56] Yang H., Marion T.N., Liu Y., Zhang L., Cao X., Hu H., Zhao Y., Herrmann M. (2019). Nanomaterial Exposure Induced Neutrophil Extracellular Traps: A New Target in Inflammation and Innate Immunity. J. Immunol. Res..

[57] Frère Y., Danicher L., Muller S., Alemán C., Bianco A., Venanzi M. (2013). Peptide Nanostructured Conjugates for Therapeutics: The Example of P140 Peptide for the Treatment of Systemic Lupus Erythematosus. Peptide Materials.

[58] Banik B.L., Fattahi P., Brown J.L. (2016). Polymeric Nanoparticles: The Future of Nanomedicine: Polymeric Nanoparticles. WIREs Nanomed Nanobiotechnol.

[59] Duncan R., Vicent M.J. (2013). Polymer Therapeutics-Prospects for 21st Century: The End of the Beginning. Adv. Drug Deliv Rev..

[60] Torchilin V.P. (2014). Multifunctional, Stimuli-Sensitive Nanoparticulate Systems for Drug Delivery. Nat. Rev. Drug Discov..

[61] Wang Y., Wen Q., Choi S.H. (2016). FDA’s Regulatory Science Program for Generic PLA/PLGA-Based Drug Products. Am. Pharm. Rev..

[62] Fromen C.A., Rahhal T.B., Robbins G.R., Kai M.P., Shen T.W., Luft J.C., DeSimone J.M. (2016). Nanoparticle Surface Charge Impacts Distribution, Uptake and Lymph Node Trafficking by Pulmonary Antigen-Presenting Cells. Nanomed. Nanotechnol. Biol. Med..

[63] Zhang Y.-N., Lazarovits J., Poon W., Ouyang B., Nguyen L.N.M., Kingston B.R., Chan W.C.W. (2019). Nanoparticle Size Influences Antigen Retention and Presentation in Lymph Node Follicles for Humoral Immunity. Nano Lett..

[64] Cruz L.J., Rosalia R.A., Kleinovink J.W., Rueda F., Löwik C.W.G.M., Ossendorp F. (2014). Targeting Nanoparticles to CD40, DEC-205 or CD11c Molecules on Dendritic Cells for Efficient CD8+ T Cell Response: A Comparative Study. J. Control. Release.

[65] Patil T.S., Deshpande A.S. (2020). Mannosylated Nanocarriers Mediated Site-Specific Drug Delivery for the Treatment of Cancer and Other Infectious Diseases: A State of the Art Review. J. Control. Release.

[66] Xiao B., Laroui H., Ayyadurai S., Viennois E., Charania M.A., Zhang Y., Merlin D. (2013). Mannosylated Bioreducible Nanoparticle-Mediated Macrophage-Specific TNF-α RNA Interference for IBD Therapy. Biomaterials.

[67] Deng F., He S., Cui S., Shi Y., Tan Y., Li Z., Huang C., Liu D., Zhi F., Peng L. (2019). A Molecular Targeted Immunotherapeutic Strategy for Ulcerative Colitis via Dual-Targeting Nanoparticles Delivering MiR-146b to Intestinal Macrophages. J. Crohns Colitis.

[68] Hu G., Guo M., Xu J., Wu F., Fan J., Huang Q., Yang G., Lv Z., Wang X., Jin Y. (2019). Nanoparticles Targeting Macrophages as Potential Clinical Therapeutic Agents Against Cancer and Inflammation. Front. Immunol..

[69] Heo R., You D.G., Um W., Choi K.Y., Jeon S., Park J.-S., Choi Y., Kwon S., Kim K., Kwon I.C. (2017). Dextran Sulfate Nanoparticles as a Theranostic Nanomedicine for Rheumatoid Arthritis. Biomaterials.

[70] Maldonado R.A., LaMothe R.A., Ferrari J.D., Zhang A.-H., Rossi R.J., Kolte P.N., Griset A.P., O’Neil C., Altreuter D.H., Browning E. (2015). Polymeric Synthetic Nanoparticles for the Induction of Antigen-Specific Immunological Tolerance. Proc. Natl. Acad. Sci. USA.

[71] Prasad S., Xu D., Miller S.D. (2012). Tolerance Strategies Employing Antigen-Coupled Apoptotic Cells and Carboxylated PLG Nanoparticles for the Treatment of Type 1 Diabetes. Rev. Diabet. Stud..

[72] Cappellano G., Woldetsadik A.D., Orilieri E., Shivakumar Y., Rizzi M., Carniato F., Gigliotti C.L., Boggio E., Clemente N., Comi C. (2014). Subcutaneous Inverse Vaccination with PLGA Particles Loaded with a MOG Peptide and IL-10 Decreases the Severity of Experimental Autoimmune Encephalomyelitis. Vaccine.

[73] Barenholz Y. (2012). (Chezy) Doxil® — The First FDA-Approved Nano-Drug: Lessons Learned. J. Control. Release.

[74] Zahednezhad F., Saadat M., Valizadeh H., Zakeri-Milani P., Baradaran B. (2019). Liposome and Immune System Interplay: Challenges and Potentials. J. Control. Release.

[75] La-Beck N.M., Liu X., Wood L.M. (2019). Harnessing Liposome Interactions With the Immune System for the Next Breakthrough in Cancer Drug Delivery. Front. Pharmacol..

[76] Maja L., Željko K., Mateja P. (2020). Sustainable Technologies for Liposome Preparation. J. Supercrit. Fluids.

[77] Ahmed K.S., Hussein S.A., Ali A.H., Korma S.A., Lipeng Q., Jinghua C. (2019). Liposome: Composition, Characterisation, Preparation, and Recent Innovation in Clinical Applications. J. Drug Target..

[78] Sercombe L., Veerati T., Moheimani F., Wu S.Y., Sood A.K., Hua S. (2015). Advances and Challenges of Liposome Assisted Drug Delivery. Front. Pharmacol..

[79] Brusini R., Varna M., Couvreur P. (2020). Advanced Nanomedicines for the Treatment of Inflammatory Diseases. Adv. Drug Deliv Rev..

[80] Metselaar J.M., Wauben M.H.M., Wagenaar-Hilbers J.P.A., Boerman O.C., Storm G. (2003). Complete Remission of Experimental Arthritis by Joint Targeting of Glucocorticoids with Long-Circulating Liposomes. Arthritis Rheum..

[81] Moallem E., Koren E., Ulmansky R., Pizov G., Barlev M., Barenholz Y., Naparstek Y. (2016). A Liposomal Steroid Nano-Drug for Treating Systemic Lupus Erythematosus. Lupus.

[82] Capini C., Jaturanpinyo M., Chang H.-I., Mutalik S., McNally A., Street S., Steptoe R., O’Sullivan B., Davies N., Thomas R. (2009). Antigen-Specific Suppression of Inflammatory Arthritis Using Liposomes. J. Immunol..

[83] Meka R.R., Venkatesha S.H., Moudgil K.D. (2018). Peptide-Directed Liposomal Delivery Improves the Therapeutic Index of an Immunomodulatory Cytokine in Controlling Autoimmune Arthritis. J. Control. Release.

[84] Poh S., Chelvam V., Kelderhouse L.E., Ayala-López W., Vaitilingam B., Putt K.S., Low P.S. (2017). Folate-Conjugated Liposomes Target and Deliver Therapeutics to Immune Cells in a Rat Model of Rheumatoid Arthritis. Nanomedicine.

[85] Galea R., Nel H.J., Talekar M., Liu X., Ooi J.D., Huynh M., Hadjigol S., Robson K.J., Ting Y.T., Cole S. (2019). PD-L1- and Calcitriol-Dependent Liposomal Antigen-Specific Regulation of Systemic Inflammatory Autoimmune Disease. JCI Insight.

[86] Bergot A.-S., Buckle I., Cikaluru S., Naranjo J.L., Wright C.M., Zheng G., Talekar M., Hamilton-Williams E.E., Thomas R. (2020). Regulatory T Cells Induced by Single-Peptide Liposome Immunotherapy Suppress Islet-Specific T Cell Responses to Multiple Antigens and Protect from Autoimmune Diabetes. J. Immunol..

[87] Pujol-Autonell I., Mansilla M.-J., Rodriguez-Fernandez S., Cano-Sarabia M., Navarro-Barriuso J., Ampudia R.-M., Rius A., Garcia-Jimeno S., Perna-Barrull D., Martinez-Caceres E. (2017). Liposome-Based Immunotherapy against Autoimmune Diseases: Therapeutic Effect on Multiple Sclerosis. Nanomedicine (Lond.).

[88] Muller S., Guichard G., Benkirane N., Brown F., van Regenmortel M.H., Briand J.P. (1995). Enhanced Immunogenicity and Cross-Reactivity of Retro-Inverso Peptidomimetics of the Major Antigenic Site of Foot-and-Mouth Disease Virus. Pept Res..

[89] Bonam S.R., Partidos C.D., Halmuthur S.K.M., Muller S. (2017). An Overview of Novel Adjuvants Designed for Improving Vaccine Efficacy. Trends Pharmacol. Sci..

[90] Friede M., Muller S., Briand J.P., Plaué S., Fernandes I., Frisch B., Schuber F., van Regenmortel M.H. (1994). Selective Induction of Protection against Influenza Virus Infection in Mice by a Lipid-Peptide Conjugate Delivered in Liposomes. Vaccine.

[91] Han J., Zhao D., Li D., Wang X., Jin Z., Zhao K. (2018). Polymer-Based Nanomaterials and Applications for Vaccines and Drugs. Polymers.

[92] Wong K.H., Lu A., Chen X., Yang Z. (2020). Natural Ingredient-Based Polymeric Nanoparticles for Cancer Treatment. Molecules.

[93] Anselmo A.C., Mitragotri S. (2015). A Review of Clinical Translation of Inorganic Nanoparticles. AAPS J..

[94] Dykman L.A., Khlebtsov N.G. (2017). Immunological Properties of Gold Nanoparticles. Chem. Sci..

[95] Dobrovolskaia M.A., Shurin M., Shvedova A.A. (2016). Current Understanding of Interactions between Nanoparticles and the Immune System. Toxicol. Appl. Pharmacol..

[96] Nguyen T.L., Choi Y., Kim J. (2019). Mesoporous Silica as a Versatile Platform for Cancer Immunotherapy. Adv. Mater..

[97] Shi J., Hedberg Y., Lundin M., Odnevall Wallinder I., Karlsson H.L., Möller L. (2012). Hemolytic Properties of Synthetic Nano- and Porous Silica Particles: The Effect of Surface Properties and the Protection by the Plasma Corona. Acta Biomater..

[98] Bagwe R.P., Hilliard L.R., Tan W. (2006). Surface Modification of Silica Nanoparticles to Reduce Aggregation and Nonspecific Binding. Langmuir.

[99] Han G., Ghosh P., Rotello V.M. (2007). Functionalized Gold Nanoparticles for Drug Delivery. Nanomedicine.

[100] Siddique S., Chow J.C.L. (2020). Gold Nanoparticles for Drug Delivery and Cancer Therapy. Appl. Sci..

[101] Feliu N., Docter D., Heine M., del Pino P., Ashraf S., Kolosnjaj-Tabi J., Macchiarini P., Nielsen P., Alloyeau D., Gazeau F. (2016). In Vivo Degeneration and the Fate of Inorganic Nanoparticles. Chem. Soc. Rev..

[102] Yeste A., Takenaka M.C., Mascanfroni I.D., Nadeau M., Kenison J.E., Patel B., Tukpah A.-M., Babon J.A.B., DeNicola M., Kent S.C. (2016). Tolerogenic Nanoparticles Inhibit T Cell-Mediated Autoimmunity through SOCS2. Sci. Signal..

[103] Raimondo T.M., Mooney D.J. (2018). Functional Muscle Recovery with Nanoparticle-Directed M2 Macrophage Polarization in Mice. Proc. Natl. Acad. Sci. USA.

[104] Wang L., Zhang H., Sun L., Gao W., Xiong Y., Ma A., Liu X., Shen L., Li Q., Yang H. (2020). Manipulation of Macrophage Polarization by Peptide-Coated Gold Nanoparticles and Its Protective Effects on Acute Lung Injury. J. Nanobiotechnol.

[105] Ventola C.L. (2017). Progress in Nanomedicine: Approved and Investigational Nanodrugs. P T.

[106] Singh P., Pandit S., Mokkapati V.R.S.S., Garg A., Ravikumar V., Mijakovic I. (2018). Gold Nanoparticles in Diagnostics and Therapeutics for Human Cancer. Int. J. Mol. Sci..

[107] Lee S.-M., Kim H.J., Ha Y.-J., Park Y.N., Lee S.-K., Park Y.-B., Yoo K.-H. (2013). Targeted Chemo-Photothermal Treatments of Rheumatoid Arthritis Using Gold Half-Shell Multifunctional Nanoparticles. ACS Nano.

[108] Kang H., Jung H.J., Kim S.K., Wong D.S.H., Lin S., Li G., Dravid V.P., Bian L. (2018). Magnetic Manipulation of Reversible Nanocaging Controls *In Vivo* Adhesion and Polarization of Macrophages. ACS Nano.

[109] Sangtani A., Nag O.K., Field L.D., Breger J.C., Delehanty J.B. (2017). Multifunctional Nanoparticle Composites: Progress in the Use of Soft and Hard Nanoparticles for Drug Delivery and Imaging. Wiley Interdiscip. Rev. Nanomed. Nanobiotechnol..

[110] Jafari S., Derakhshankhah H., Alaei L., Fattahi A., Varnamkhasti B.S., Saboury A.A. (2019). Mesoporous Silica Nanoparticles for Therapeutic/Diagnostic Applications. Biomed. Pharmacother.

[111] FDA (2019). Food and Drug Administration Code of Federal Regulations Title 21.

[112] Benezra M., Penate-Medina O., Zanzonico P.B., Schaer D., Ow H., Burns A., DeStanchina E., Longo V., Herz E., Iyer S. (2011). Multimodal Silica Nanoparticles Are Effective Cancer-Targeted Probes in a Model of Human Melanoma. J. Clin. Investig..

[113] Chen W., Glackin C.A., Horwitz M.A., Zink J.I. (2019). Nanomachines and Other Caps on Mesoporous Silica Nanoparticles for Drug Delivery. Acc. Chem. Res..

[114] Zheng X., Zeng S., Hu J., Wu L., Hou X. (2018). Applications of Silica-Based Nanoparticles for Multimodal Bioimaging. Appl. Spectrosc. Rev..

[115] Maggini L., Cabrera I., Ruiz-Carretero A., Prasetyanto E.A., Robinet E., de Cola L. (2016). Breakable Mesoporous Silica Nanoparticles for Targeted Drug Delivery. Nanoscale.

[116] Maggini L., Travaglini L., Cabrera I., Castro-Hartmann P., de Cola L. (2016). Biodegradable Peptide-Silica Nanodonuts. Chemistry.

[117] Picchetti P., DiMarco B.N., Travaglini L., Zhang Y., Ortega-Liebana M.C., de Cola L. (2020). Breaking with Light: Stimuli-Responsive Mesoporous Organosilica Particles. Chem. Mater..

[118] Stead S.O., Kireta S., McInnes S.J.P., Kette F.D., Sivanathan K.N., Kim J., Cueto-Diaz E.J., Cunin F., Durand J.-O., Drogemuller C.J. (2018). Murine and Non-Human Primate Dendritic Cell Targeting Nanoparticles for *in Vivo* Generation of Regulatory T-Cells. ACS Nano.

[119] Gan J., Dou Y., Li Y., Wang Z., Wang L., Liu S., Li Q., Yu H., Liu C., Han C. (2018). Producing Anti-Inflammatory Macrophages by Nanoparticle-Triggered Clustering of Mannose Receptors. Biomaterials.

[120] Zhang X., Zhang X., Wang X., Wang T., Bai B., Zhang N., Zhao Y., Yu Y., Wang B. (2020). Efficient Delivery of Triptolide Plus a MiR-30-5p Inhibitor Through the Use of Near Infrared Laser Responsive or CADY Modified MSNs for Efficacy in Rheumatoid Arthritis Therapeutics. Front. Bioeng. Biotechnol..

[121] Kim J., Kim H.Y., Song S.Y., Go S.-H., Sohn H.S., Baik S., Soh M., Kim K., Kim D., Kim H.-C. (2019). Synergistic Oxygen Generation and Reactive Oxygen Species Scavenging by Manganese Ferrite/Ceria Co-Decorated Nanoparticles for Rheumatoid Arthritis Treatment. ACS Nano.

[122] Liu Z., Chen X., Zhang Z., Zhang X., Saunders L., Zhou Y., Ma P.X. (2018). Nanofibrous Spongy Microspheres To Distinctly Release MiRNA and Growth Factors To Enrich Regulatory T Cells and Rescue Periodontal Bone Loss. ACS Nano.

[123] Palmer B.C., Jatana S., Phelan-Dickinson S.J., DeLouise L.A. (2019). Amorphous Silicon Dioxide Nanoparticles Modulate Immune Responses in a Model of Allergic Contact Dermatitis. Sci. Rep..

[124] De la Escosura-Muñiz A., Parolo C., Merkoçi A. (2010). Immunosensing Using Nanoparticles. Mater. Today.

[125] Jane A.O., Szili E.J., Reed J.H., Gordon T.P., Voelcker N.H., Nicolau D.V., Abbott D., Kalantar-Zadeh K., di Matteo T., Bezrukov S.M. (2007). Porous Silicon Biosensor for the Detection of Autoimmune Diseases.

[126] Niemeyer C.M. (2007). Functional Devices from DNA and Proteins. Nano Today.

[127] Wang J., Qu X. (2013). Recent Progress in Nanosensors for Sensitive Detection of Biomolecules. Nanoscale.

[128] Liu G., Lovell J.F., Zhang L., Zhang Y. (2020). Stimulus-Responsive Nanomedicines for Disease Diagnosis and Treatment. IJMS.

[129] Mura S., Nicolas J., Couvreur P. (2013). Stimuli-Responsive Nanocarriers for Drug Delivery. Nat. Mater..

[130] Licciardello N., Hunoldt S., Bergmann R., Singh G., Mamat C., Faramus A., Ddungu J.L.Z., Silvestrini S., Maggini M., de Cola L. (2018). Biodistribution Studies of Ultrasmall Silicon Nanoparticles and Carbon Dots in Experimental Rats and Tumor Mice. Nanoscale.

[131] Baeza A., Vallet-Regí M. (2020). Mesoporous Silica Nanoparticles as Theranostic Antitumoral Nanomedicines. Pharmaceutics.

[132] Kopansky-Groisman E., Kogan-Zviagin I., Sella-Tavor O., Oron-Herman M., David A. (2019). Near-Infrared Fluorescent Activated Polymeric Probe for Imaging Intraluminal Colorectal Cancer Tumors. Biomacromolecules.

[133] Pola R., Böhmová E., Filipová M., Pechar M., Pankrác J., Větvička D., Olejár T., Kabešová M., Poučková P., Šefc L. (2020). Targeted Polymer-Based Probes for Fluorescence Guided Visualization and Potential Surgery of EGFR-Positive Head-and-Neck Tumors. Pharmaceutics.

[134] Janisova L., Gruzinov A., Zaborova O.V., Velychkivska N., Vaněk O., Chytil P., Etrych T., Janoušková O., Zhang X., Blanchet C. (2020). Molecular Mechanisms of the Interactions of N-(2-Hydroxypropyl)Methacrylamide Copolymers Designed for Cancer Therapy with Blood Plasma Proteins. Pharmaceutics.

[135] Lim M.S.H., Ohtsuki T., Takenaka F., Kobayashi K., Akehi M., Uji H., Kobuchi H., Sasaki T., Ozeki E., Matsuura E. (2021). A Novel 89Zr-Labeled DDS Device Utilizing Human IgG Variant (ScFv): “Lactosome” Nanoparticle-Based Theranostics for PET Imaging and Targeted Therapy. Life.

[136] Bhaskaran S., Sharma N., Tiwari P., Singh S.R., Sahi S.V. (2019). Fabrication of Innocuous Gold Nanoparticles Using Plant Cells in Culture. Sci. Rep..

[137] Getts D.R., Shea L.D., Miller S.D., King N.J.C. (2015). Harnessing Nanoparticles for Immune Modulation. Trends Immunol..

[138] Bianco A., Muller S. (2016). Nanomaterials, Autophagy, and Lupus Disease. ChemMedChem.

